# Assessment of disease named entity recognition on a corpus of annotated sentences

**DOI:** 10.1186/1471-2105-9-S3-S3

**Published:** 2008-04-11

**Authors:** Antonio Jimeno, Ernesto Jimenez-Ruiz, Vivian Lee, Sylvain Gaudan, Rafael Berlanga, Dietrich Rebholz-Schuhmann

**Affiliations:** 1 European Bioinformatics Institute, Wellcome Trust Genome Campus, Hinxton, Cambridge, CB10 1SD, UK; 2 Departamento de Lenguajes y Sistemas Informaticos, Universitat Jaume I, Castellon de la Plana, 12071, Spain

## Abstract

**Background:**

In recent years, the recognition of semantic types from the biomedical scientific literature has been focused on named entities like protein and gene names (PGNs) and gene ontology terms (GO terms). Other semantic types like diseases have not received the same level of attention. Different solutions have been proposed to identify disease named entities in the scientific literature. While matching the terminology with language patterns suffers from low recall (e.g., Whatizit) other solutions make use of morpho-syntactic features to better cover the full scope of terminological variability (e.g., MetaMap). Currently, MetaMap that is provided from the National Library of Medicine (NLM) is the state of the art solution for the annotation of concepts from UMLS (Unified Medical Language System) in the literature. Nonetheless, its performance has not yet been assessed on an annotated corpus. In addition, little effort has been invested so far to generate an annotated dataset that links disease entities in text to disease entries in a database, thesaurus or ontology and that could serve as a gold standard to benchmark text mining solutions.

**Results:**

As part of our research work, we have taken a corpus that has been delivered in the past for the identification of associations of genes to diseases based on the UMLS Metathesaurus and we have reprocessed and re-annotated the corpus. We have gathered annotations for disease entities from two curators, analyzed their disagreement (0.51 in the kappa-statistic) and composed a single annotated corpus for public use. Thereafter, three solutions for disease named entity recognition including MetaMap have been applied to the corpus to automatically annotate it with UMLS Metathesaurus concepts. The resulting annotations have been benchmarked to compare their performance.

**Conclusions:**

The annotated corpus is publicly available at  and can serve as a benchmark to other systems. In addition, we found that dictionary look-up already provides competitive results indicating that the use of disease terminology is highly standardized throughout the terminologies and the literature. MetaMap generates precise results at the expense of insufficient recall while our statistical method obtains better recall at a lower precision rate. Even better results in terms of precision are achieved by combining at least two of the three methods leading, but this approach again lowers recall. Altogether, our analysis gives a better understanding of the complexity of disease annotations in the literature. MetaMap and the dictionary based approach are available through the Whatizit web service infrastructure (Rebholz-Schuhmann D, Arregui M, Gaudan S, Kirsch H, Jimeno A: Text processing through Web services: Calling Whatizit. *Bioinformatics* 2008, 24:296-298).

## Background

The literature provides information that is not covered in other knowledge resources, for example in thesauri, databases or ontologies. In the biomedical domain the integration and transfer of knowledge from textual sources to these other knowledge sources is ongoing work and requires a lot of effort. An example of such a resource that profits in particular from this transfer is the OMIM (Online Mendelian Inheritance in Man) database [1]. The transfer of information though suffers from the high growth rate in literature information and publication numbers that turns manual curation into a rather slow process. Hence, researchers in text mining focus on the automation of this process that profits from different analytical steps, for example low level natural language processing or recognition of named entities.

Term recognition deals with the identification of relevant terms and term resolution intends to link these terms to entries in existing knowledge sources (e.g., a database entry). Although quite some effort has been spent on the identification of protein and gene named entities (PGNs) in the scientific literature, little work has been invested on the recognition and resolution of other terminologies in the biomedical domain. This is partly due to the fact that annotated corpora are not abundant due to the high costs attached to their generation.

The BioCreAtIvE Challenge I and II [2] have tackled the gene recognition and resolution tasks apart from the identification of Gene Ontology [3] terms and for both challenges an annotated corpus has been generated. The NLPBA challenge [4] has been based on the Genia Corpus [5] that contains annotations of concepts from the Genia ontology. Some work has been devoted to the identification of chemical entities [6,7] linked to chemical databases like PubChem [8] or ChEBI [9]. All these efforts are tied up with the generation of corpora that help to improve existing named entity recognition solutions that eventually become available to the general public (as Abner [10] or LingPipe [11]). Furthermore, such solutions can be used in other tasks like information retrieval as has been shown in the TREC Genomics Track [12]. Solutions for the identification of disease named entities have been made publicly available, such as MetaMap [13] or selected modules in Whatizit [14], but any of them still lack proper assessment against a gold standard.

The recognition of disease terms in the literature is relevant to identify known and hypothetical causes of the disease such as genes that are involved in a disease as well as known treatments of a disease such as drugs, chemicals or chemical compounds. OMIM is an example of a database that is based on information from the literature, i.e. it has been generated by manual curation of scientific papers. An automatic analysis of the literature could expedite the population of this database.

Some research work has been done on the identification of diseases: refer to the work of Craven [15,16], Krallinger [17] and Chun [18] for the identification of relations between disease entities and PGNs. In the first work, the diseases where identified automatically using the existing OMIM lexicon. In the second, the authors provide a set of sentences together with the mentions of genes and diseases but the corpus does not deliver the annotations of the diseases as part of the sentence. In the third work, the disease and PGNs were identified in a random selection from a subset of Medline using a look-up technique based on the UMLS, the assessment was done at the level of NER but the resolution of the term into the proper UMLS entry was not done. As a conclusion, there is no gold standard that can be used to do the preparation and evaluation of existing techniques focused on the identification of diseases in text.

Several terminological resources are available that provide disease terms. Amongst the most used resources are the Medical Subject Headings (MeSH) [19], the NCI (National Cancer Institute) thesaurus [20], Snomed CT [21] and finally the UMLS resource [22] that encloses all the other ones into a single source. MeSH and Snomed CT have a different scope and fulfill different tasks and none of them has been designed to meet text mining needs. Since we are interested on the recognition of diseases in text, UMLS is a natural choice since it is covering more than the other sources.

In this paper, we benchmark three different methods that make use of the UMLS Metathesaurus for disease entity identification. All measured results are based on an annotated corpus that is publicly available from our web site. Our measurements result from complementary methods, i.e. from a lexical approach (dictionary look-up method), from an information theoretical approach (statistical approach) and from MetaMap (a morpho-syntactical heuristics with a lexicon). In addition, we measured the performance of combinations of all three methods. These results give a better understanding of the complexity of the task and the appropriateness of the methods applied.

## Results and discussion

Our analysis leads into two different assessments. The first one is based on entity recognition. In this task we analyze how all methods perform on the identification of the boundaries of the disease mentions in the text. This task is similar to the Gene Mention task in BioCreAtIvE. The second task focuses on the recognition of diseases at the sentence level. Here, we want to assess the correct resolution of disease terms. Since the recognition of these terms may not be limited to the boundaries of a noun phrase, we have decided to select the sentence boundaries as the minimum stretch of text that would be relevant for annotations of diseases. This task is similar to the Gene Normalization task in BioCreAtIvE. The evaluation of the applied methods requires to define the benchmark that is used for the assessment (i.e. the annotated corpus) and evaluation measures, for example precision, recall and F-measure that are well known standards (ref. to Methods section).

### NER evaluation

In our research work we wanted to assess the recognition and resolution of disease named entities from natural language text. We identified the corpus provided by Craven [16] as relevant to our evaluation, although it has the limitation of being restricted to OMIM diseases only.

Typically all NER evaluations consider correct matching of both, the left and the right boundary of the named entity as the most precise assessment (called *exact matching*). Others have proposed to deviate from exact matching: As shown in [23] more flexible matching of the boundaries in the evaluation produces better results and may be more appropriate in the evaluation. We assessed our results as well against exact matching as well against the match of the left boundary and right boundary only, respectively. The results are shown in table 1. In all three cases, the dictionary look-up performed best and in particular the assessment against an alignment to the right boundary showed best results, where general disease terms such as *malignant cancer* were accepted as a correct match against the underlying disease evidence such as *familial malignant neoplasm*. This example shows the overall better results for right alignment in comparison to the other two assessments in the case of dictionary look-up. Our statistical method and MetaMap operate on regions defined by their NLP processes that do not necessarily comply to a defined chunk such as a noun phrase. This leads to the condition that the boundaries of the text evidence used for the term recognition do not match at either end exactly to the boundaries of the term representation in the terminological resource.

**Table 1:** NER evaluation. This table shows the result of NER. For each alingment and method, first we show how many diseases are annotated in the corpus (OMIM) then the total number of annotation done by the method and then how many of them agreed with the benchmark (TP).

		OMIM	Annotated	TP	Precision	Recall	F-measure
Exact boundary	D look-up	908	1063	584	**54.94**	**64.32**	**59.26**
	Statistical	908	1051	274	26.07	30.18	27.97
	MetaMap	908	873	273	31.27	30.07	30.66
	D look-up	908	1063	598	**56.26**	**65.86**	**60.68**
	Statistical	908	1051	390	39.86	38.33	39.08
Left alignment	MetaMap	908	873	348	37.11	42.95	39.82
Right alignment	D look-up	908	1063	685	**64.44**	**75.44**	**69.51**
	Statistical	908	1051	439	53.49	51.43	52.44
	MetaMap	908	873	467	41.77	48.35	44.82

The low performance of the applied methods is partly explained by the mismatch between the terminology used by Craven to do the annotation of diseases and our terminology (described in the Methods section). We have identified additional terms that are occurrences of diseases in text but are not identified as such in Craven's data set due to missing mentions of the disease in the OMIM lexicon such as *retinoblastoma* or terms not annotated because a given disease term represents a term variant in comparison to corresponding entry in OMIM's such as *anhaptoglobinemia*, *del* versus *anhaptoglobinemia*. In a similar work [18] it has been proved that filtering techniques based on machine learning can improve the performance of look-up techniques. In similar tasks but for different semantic types, as PGNs, machine learning techniques like SVM or conditional random fields are the state of the art. Further research is needed to assess these techniques on disease recognition.

### Term recognition evaluation

In this task we want to link the diseases with the appropriate concept in the UMLS data resource. The evaluation consists in comparing the annotations produced by each one of the methods with the annotations in the gold standard. According to our assumptions, the occurrence of a representation of a disease term is limited to the sentence boundaries.

All annotations of disease entities have been collected at the sentence level for all three applied methods to better compare the results. All three methods use different local contexts in their ways to match text evidence to the representation of a disease named entity, i.e. the mention of the disease is not always limited to a noun phrase as used in the dictionary look-up. Our statistical approach can be adjusted to analyze a zone that is larger than the noun phrases up to the stretch of a complete sentence. MetaMap delivers annotations in a local context that resembles the stretch of a noun phrase but tends to be larger than the typical noun phrase definition, for example MetaMap extends the noun phrase with punctuation extensions. All annotations of all methods have been collected at the sentence level.

As no corpus is available that can serve as a gold standard we have processed the available corpus consisting of a set of sentences. After the annotation of the results with all three methods, two domain experts have analyzed the results. For more details refer to the Methods section.

Table 2 shows the results obtained from the different methods. We can see that the statistical method has the highest recall, MetaMap obtains the best performance in terms of precision and the dictionary look-up has the highest F-measure.

**Table 2:** Recognition of disease in sentences. This table shows the total number of diseases in the benchmark (Benchmark), the number of distinct diseases (Diseases), the number of diseases annotated by the method and the number of unique diseases identified. Then we present the result on standard measures like precision and recall.

	Benchmark	Diseases	Annotated	Diseases	FP	TP	Recall	Precision	F-measure
D look-up	924	280	699	226	144	555	60.06	79.40	**68.39**
Statistical	924	280	937	309	317	620	**67.10**	66.17	66.63
MetaMap	924	280	590	192	95	495	53.57	**83.90**	65.39
Vote 1	924	280	1164	358	298	866	**93.72**	74.40	**82.95**
Vote 2	924	280	696	228	124	572	61.90	82.18	70.62
Vote 3	924	280	388	137	30	358	38.74	**92.27**	54.57

**Figure 1:**
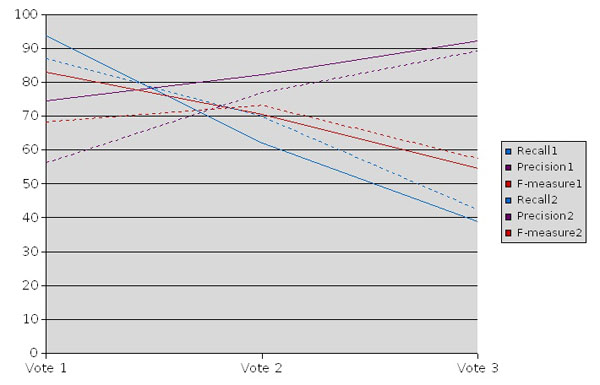
**Recognition of diseases in sentences based on voting**. This figure compares the performance of the different voting combinations and the voting after removing the most frequent diseases (in dotted lines).

In addition we combine the results from all three different methods by a voting scheme. In the first approach, we picked up any disease that has been proposed by at least one of all methods. In the second and third approach we required that at least two methods agree or all three of them. The first combination of our methods obtains the highest recall showing that all three methods are complementary. If we require a higher level of agreement between the methods, we see an increase in the precision but at the cost of lower recall (ref. to figure 1).

We found that selected diseases appear at high frequencies in the corpus and are most likely overrepresented. This could be the reason for a bias in our assessment, since the overall measurements could be mainly based on the occurrence of the most frequent diseases in the corpus. This would lead to the consequence that the observed differences between the methods are due to the behavior that they show on the basis of the frequent disease mentions. We defined frequent diseases as the ones with more than 15 occurrences in the benchmark corpus. The list of theses diseases can be found in table 3. In order to generate more representative results, we have removed these diseases from our test data and have reassessed the performance of our systems. The results are presented in table 4. As we can read from the result table, we reduced the overall number of disease annotations in our result set by around 36% although we removed only 11 diseases (4% of the diseases). Although the figures for precision and recall have been lower now, the differences overall between all three methods is still in the same range.

**Table 3:** Most frequent terms in the disease recognition benchmark

Frequency	Term
67	Ovarian cancer
58	Breast carcinoma
51	Glucose-6-phosphate dehydrogenase deficiency anemia
26	Myotonic dystrophy
26	Muscular dystrophy, duchenne
21	Enzyme deficiency
20	Adrenoleukodystrophy, Neonatal
20	Aniridia
17	Hereditary breast/ovarian cancer (BRCA1, BRCA2)
17	Familial cancer of breast
15	Phenylketonurias

**Table 4:** Recognition of disease in sentences; without the most frequent diseases. This table shows the total number of diseases in the benchmark (Benchmark), the number of distinct diseases (Diseases), the number of diseases annotated by the method and the number of unique diseases identified. Then we present the result on standard measures like precision and recall.

	Benchmark	Diseases	Annotated	Diseases	FP	TP	Recall	Precision	F-measure
D look-up	586	269	542	217	143	399	68.09	73.62	**70.74**
Statistical	586	269	760	299	313	447	**76.28**	58.82	66.42
MetaMap	586	269	413	182	95	318	54.27	**77.00**	63.66
Vote 1	586	269	908	347	398	510	**87.03**	56.17	68.27
Vote 2	586	269	532	218	123	409	69.80	76.88	**73.17**
Vote 3	586	269	278	130	30	248	42.32	**89.21**	57.41

## Discussion

As we can observe from our analysis, the results of the dictionary look-up and from the statistical approach are in the same range as the performance of MetaMap in terms of F-measure. We can expect that the results could be further improved, if at least the statistical approach is better fitted to the data in the gold standard corpus.

All methods require improvements regarding the recall and looking into the causes for low recall we find the following issues for improvement. Recall is limited by the fact that not all lexical variants found in the text are contained in the UMLS Metathesaurus (e.g., *complement 6 deficiency* versus *c6 deficiency*).

Complex syntactic variants like coordination (*breast and colon cancer*) are difficult to capture and are common to the recognition of semantic types such as PGNs [24].

Low precision is due to false positives that are generated from different findings. Some methods have proposed general terms like *neoplasm* that did not fit the full form in the text. On the other side, we encountered the prediction of very specific terms, for example *primary malignant neoplasm* versus *malignant neoplasm*, that were not represented in the text. In addition, our methods proposed related terms that still represented different semantic types such as *von Hippel-Lindau syndrome* versus *von Hippel-Lindau mutation* or had to cope with ambiguity. For example *retinoblastoma* was proposed as disease, but was referred to in the text as a gene. This phenomenon has already been discussed by Chen et al. [25] in their study about ambiguity of PGNs. A very common source of ambiguity is the reuse of acronyms like EMD for different senses, for example *Emery-Dreifuss muscular dystrophy* and for *electromechanical dissociation *[26]. Finally, some redundant and ambiguous terms are contained in the UMLS Metathesaurus lexicon itself, despite all the filtering applied to possible ambiguous terms (for example *Sensitivity of* as a synonym of *Dentin sensitivity*).

The combination of all three methods to propose annotations leads to modified precision and recall characteristics. If we accept all annotations that have been proposed by at least one method the recall increases. This shows that all three methods are complementary and recognize diseases that have not been recognized by the other two methods. The contrary is true, if two of the three or all three methods have to agree on the annotation. In this case the precision increases and at the same time the recall is lower. This again shows that all three methods are complementary and make different assumptions concerning the recognition of diseases in the literature.

We have further analyzed the results under the condition that two of the methods agree but the third one disagrees. We find altogether 216 cases and in 173 of these cases the disagreement results from MetaMap. This shows that the statistical approach and the dictionary look-up have more similar performances than MetaMap. Only in 148 cases all three methods agreed and all three methods disagreed in 164 cases.

These results can also be read from the measurement of the overall performance of the methods in tables 2 and 4. In the dictionary look-up approach terms are matched to the lexicon of the concepts giving priority to the longest span of text that matches the entity; some annotations may refer to occurrences of the term in nested terms. No variation is allowed so it may present higher precision at the cost of lower recall; as we will show with the statistical method. Analyzing the results we find that with this simple method we obtain a result that performs better than the other two methods considering the F-measure. This means that use of disease terms in text is well standardized. The statistical approach obtains the highest recall this is not a surprising result since the matching of terms is more flexible than in the dictionary look-up. The recall is not significantly much higher than the dictionary look-up, further supporting the assumption that the disease terminology is quite close to its usage in text. MetaMap relies on morpho-syntactic processing of terms and text and selects a region of the sentence that is larger than the occurrence of the disease term in text. This is the cause to the finding that in some cases the score provided by MetaMap is lower than other terms selected by MetaMap. In addition, at times the occurrence of a disease term exceeds the window selected by the NLP processing tools used by MetaMap.

## Conclusions

The corpus with the curated annotations is available at [27] and can be used to optimize the parameters of a different system performing disease annotations. The corpus allowed us to assess different methods. It covers already a significant number of disease entities and accounts for a selection of term variability problems that are encountered in the recognition of disease entities in the literature. The corpus enables research teams to develop other systems for the recognition of diseases.

The comparison of the approaches applied on this corpus shows that dictionary look-up provides already competitive results. We read from this finding that the disease terminology is well covered in the available terminological resources and well standardized in the literature. Furthermore, we observe and explain phenomena in the identification of disease terms that are better addressed by one or the other applied methods. These phenomena may not apply to other semantic types such as GO (Gene Ontology) terms or PGNs. For instance GO terms are longer than disease terms and the purpose of GO is not meant for text mining, so it is harder to identify them in text as such using a lexical look-up solution. PGNs on the other hand can be recognized with high recall in text with a look-up approach but require a special treatment due to the ambiguity of the terms with common English, other semantic types as disease or other PGNs belonging to different species.

The results show some directions for further research. Concerning the recall, we can see that the lexicon is quite extensive but some lexical variants can be found that are not covered by the current resources.

Morphological variants may be considered and may be dependent of the semantic type covered; the same analysis cannot be applied to PGNs. On the other hand, syntactic variants like coordination have already been studied [28] but are not intensively considered in the biomedical domain and an interest has already been shown for PGN recognition in the BioCreAtIvE Challenge [29]. Some tools already exist that do term normalization like the SPECIALIST lexical [30] tools available from the NLM. Concerning precision we may find common issues concerning the current approaches that relate to the disambiguation of the term to the appropriate semantic type for which resources like the UMLS Project (absolutely relevant but needs further research) and the disambiguation of abbreviations that is already one research topic in many research groups.

## Methods

In the literature we can identify several techniques to do the mapping between terms and data sources that go from dictionary look-up to more sophisticated techniques based on natural language processing and machine learning; as can be seen for example in the Gene Normalization task in BioCreAtIvE [29]. We propose to use three approaches that rely on different underlying technologies. A dictionary look-up approach, a statistical approach (information theoretical approach) and MetaMap (the state of the art annotation application). Since no gold standard dataset was available for the recognition of disease named entities, no machine learning technique has been used yet. The different methods share the same data source, the UMLS Metathesaurus to do the mapping. MetaMap already includes its own processing of the UMLS Metathesaurus. We have processed it as well and the outcome is used by the dictionary look-up and the statistical method. The processing performed on the UMLS Metathesaurus is explained in the following section.

### UMLS Metathesaurus

The UMLS Metathesaurus [31] is one of the three components of the UMLS Project and comprises many different controlled vocabularies. Each contained concept is linked to a set of synonyms similar to the data structures used in WordNet. In addition, it provides relations between different concepts like taxonomic relations and relations provided by the UMLS Semantic Network. The UMLS Semantic Network is an additional component in the UMLS Project and defines around 135 semantic types for term categorization. These types are organized in a hierarchical structure. Our UMLS version used in this project was issued in 2006AD. The preparation of the lexicon requires a number of steps [32]. The first one is to select the sources that our local installation of UMLS will contain. Then the lexicon related to the diseases has to be extracted and filtered since some terms may be too ambiguous or not representative of the disease in text, thus redundant.

Different sources contribute to UMLS. Some of these sources contain terms that are less relevant for term recognition such as numbers or single letters. We filtered the UMLS Metathesaurus Lexicon according to the steps proposed by Aronson [33]. In particular we selected only the subpart of UMLS that covers the English language. Other entries have been filtered out since they are tagged as being *Obsolete content* (flag 'O'), *Non-obsolete content but marked suppressible by an editor* (flag 'E') or *Non-obsolete content but deemed suppressible during inversion* (flag 'Y'). Concept terms with ten or more words have been deleted, since we believe that they are not used in scientific literature and thus their information content is very low for this study. Finally, we collected general terms in a stop word list that includes terms like *disease* or *syndrome* that do not to provide new information.

We also removed terms contained in parenthesis and attached to the lexical item, e.g., *Neoplasm of abdomen (disorder)*. With regards to ambiguity, the Metathesaurus has also been processed to solve some ambiguous cases, that is, strings that have two or more assigned CUIs (Concept Unique Identifier). We can distinguish three other types of term ambiguity. The first one is discussed by Aronson and Shooshan [34]. They present a set of ambiguous concept names with degree 40 to degree 6 (the degree is the number of different CUIs associated to the same term string). We have followed their work in order to detect and delete the suppressible ambiguous cases. The second case of ambiguity is the one involving concepts with different semantic types and not covered in the previous method. Cases like *brain* as a synonym of *brain disease* are representatives of this type of ambiguity. Priority was given to semantic types relevant to this project. The third case of ambiguity will involve concepts from the same semantic type. This ambiguity is not solved, and the term will have associated a set of CUIs, that is, if the string is detected in a text it will be tagged with all related CUIs; as in prostate cancer (C0600139,C0376358).

Finally the terminology has been filtered semantically to select the sources for the disease lexicon. This is done based on the UMLS semantic types. We have selected terms belonging to the following semantic types: *Disease or Syndrome*, *Neoplastic Process*, *Congenital Abnormality*, *Mental or Behavioral Dysfunction*, *Experimental Model of Disease* and *Acquired Abnormality*.

After all the processing was done, we had selected a set of 275,000 terms that can be mapped to 85,000 concepts from the different semantic types selected. This provides an average of three terms per concept.

### Dictionary look-up method

The dictionary look-up performs exact matching between the terms in the lexicon and the terms in the literature. This method matches the term as it appears in the terminology so is not robust against term variability that has not been foreseen on the creation of the lexicon. In addition, the precision may be affected by ambiguous terms or nested terms and some techniques are proposed to solve these issues [35]. We have applied finite state automata (monq package [36]) for matching of large terminology sets in text to avoid efficiency problems.

### Statistical method

This second approach [37] is an information-theory based approach that is supposed to provide a higher recall than the look-up technique. The lexicon was again process to obtain properties for all lexical items, such as the frequency of individual words in the terminological set. For every term the specificity of the term was calculated based on the information content of its tokens. When processing the text, several parameters were measured on a given zone in the text (a zone can be a noun phrase, a window or the sentence). One parameter is the evidence for a term that has been calculated from the number of tokens that have been matched in the text of the zone. Another parameter is the proximity between all the words matched in the zone measured by the distance of the indices of the individual words. All three parameters are combined in a scoring function (the product of all individual measures). The approach has been developed and tuned for the identification of GO terms and has provided better results than related techniques. For more details refer to Gaudan [37].

### MetaMap

The third method is based on MetaMap [13]. This solution is well known for the annotation of the literature with UMLS concepts. MetaMap splits the given input text into sentences, and the set of sentences into phrases. For each phrase MetaMap identifies possible matchings based on lexical look-up and on variants, from their filtered UMLS Methatesaurus [33], associating a score to each one of them. That is the reason for which MetaMap identifies several possible matchings in each phrase and several candidates for each one. However, due to its flexibility, in some cases the text matched is not fully precise with respect the UMLS concept and may extend the boundaries of the entity as we show in the evaluation of the named entities in the results section.

### Benchmark preparation

We need a benchmark for the evaluation of the different methods and to identify the specific issues that need to be addressed. As said before, there are no corpora available to perform the recognition of diseases in text. In this section we explain how we prepared our benchmark for disease recognition. The corpus contains the sentences and disease annotation at the sentence level based on UMLS disease identifiers. A distinction has to be done between the corpus used for NER and for disease recognition. The first one is based on the corpus available from Craven and the later is described in this section.

This corpus is based on a selection of sentences from Craven's corpus that is a sub-collection of OMIM. From this corpus 600 sentences have been selected. The preparation of the corpus followed two steps. In the first step the corpus is pre-annotated and in the latter the annotation has been reviewed by two domain experts.

The corpus has been pre-annotated by all before-mentioned methods (high recall) by taking any annotation provided by any of the systems. The main goal is to reduce the overhead needed to check evidence in the text against the UMLS Metathesaurus to obtain the identifier.

After the assessment, the kappa statistic between the different assessors is 0.51. This agreement is rather low and is best explained by the fact that the curators only relied on their domain knowledge, i.e. no strict guidelines were provided initially. The following issues were tracked and solved and could justify the discrepancies between the assessors.

In some cases the pre-annotated corpus was offering the choice between a more general and more specific concept, but in the text we found mentions only of the most specific one. In some cases one of the assessors chose both of them as valid annotations; this accounts for the biggest number of disagreements between the assessors. This discrepancy has been solved by selecting the closest concept matching the term in the sentence.

In other cases a more general and more specific mention of the disease happened in the sentence and in the annotation. In this case both have to appear in the benchmark since both are mentioned in text, e.g. *gm1-gangliosidosis is a lysosomal storage disorder* … mention both *gangliosidoses* and *lysosomal storage disorder*.

Another issue is that different diseases from UMLS can be assigned to the same disease evidence in the text (see above). In this case UMLS does not provide any support to take a decision for one or the other (e.g., *breast carcinoma* and *malignant cancer of the breast*).

In some cases the assessors have identified missing diseases; in other cases they identified false negative results with regard to all three methods. On the other hand, some of them were not considered as diseases by the UMLS so none of the methods considered these terms that belong to the categories *Sign or Symptom* like *diarrhea*; table 5 contains the list of these terms. This shows that the criteria to classify a concept as a disease or symptom is blur in some cases. Finally 313 different concepts from the UMLS are annotated from which mainly one term is used, meaning that around 350 terms are used.

**Table 5:** Terms provided by the curators not found in our term selection. The following terms are identified by the curators as candidates for a disease but they are assigned to a different semantic type in the UMLS than the set that we have selected

CUI	Term	Semantic Type
C0003862	Arthralgia	Sign or Symptom
C0011071	Sudden death	Finding / Pathologic Function
C0011991	Diarrhea	Sign or Sympton
C0018790	Cardiac arrest	Pathologic Function
C0019054	Haemolysis	Cell Function
C0026838	Spasticity	Sign or Sympton
C0040053	Thrombosis	Pathologic Function
C0085298	Sudden cardiac death	Pathologic Function
C0264202	Somatic dysfunction	Finding
C1257806	Chromosomal instability	Cell or Molecular Dysfunction
C1384666	Hearing impairment	Finding

## Competing interests

The authors declare that they have no competing interests.

## Authors' contributions

AJ carried out the experiments, participated in the development of the methods and drafted the manuscript. EJ carried out the implementation of the methods and drafted the manuscript. VL assessed the annotation and helped to draft the manuscript. SG carried out the implementation of the methods and helped to draft the manuscript. RB participated in design of the experiments and helped to draft the manuscript. DRS participated in design of the experiments, assessed the annotation and drafted the manuscript. All authors read and approved the final manuscript.
